# 3D Digital Modeling of Dental Casts from Their 3D CT Images with Scatter and Beam-Hardening Correction

**DOI:** 10.3390/s24061995

**Published:** 2024-03-21

**Authors:** Mohamed A. A. Hegazy, Myung Hye Cho, Min Hyoung Cho, Soo Yeol Lee

**Affiliations:** 1 R&D Center, Ray, Seongnam 13494, Republic of Korea; hegazy@khu.ac.kr (M.A.A.H.); myunghye.cho@raymedical.co.kr (M.H.C.); 2 Department of Biomedical Engineering, Kyung Hee University, Yongin 17104, Republic of Korea; mhcho@khu.ac.kr

**Keywords:** dental 3D modeling, CBCT, dental casts, scatter artifacts, beam-hardening artifacts

## Abstract

Dental 3D modeling plays a pivotal role in digital dentistry, offering precise tools for treatment planning, implant placement, and prosthesis customization. Traditional methods rely on physical plaster casts, which pose challenges in storage, accessibility, and accuracy, fueling interest in digitization using 3D computed tomography (CT) imaging. We introduce a method that can reduce both artifacts simultaneously. To validate the proposed method, we carried out CT scan experiments using plaster dental casts created from dental impressions. After the artifact correction, the CT image quality was greatly improved in terms of image uniformity, contrast-to-noise ratio (CNR), and edge sharpness. We examined the correction effects on the accuracy of the 3D models generated from the CT images. As referenced to the 3D models derived from the optical scan data, the root mean square (RMS) errors were reduced by 8.8~71.7% for three dental casts of different sizes and shapes. Our method offers a solution to challenges posed by artifacts in CT scanning of plaster dental casts, leading to enhanced 3D model accuracy. This advancement holds promise for dental professionals seeking precise digital modeling for diverse applications in dentistry.

## 1. Introduction

Three-dimensional (3D) digital dental modeling has revolutionized dentistry by providing accurate morphological representations of the maxilla, mandible, and teeth. Digital modeling allows for precise dental treatment planning, implant placement, and customized prosthetics. One of the prevalent 3D dental modeling methods is the use of 3D computed tomography (CT) images of plaster dental casts [1,2,3]. Plaster dental casts have long been used in dentistry as a physical representation of a patient’s teeth and bone structure. In general, plaster dental casts of adults do not have significant variance in size and shape [4]. Plaster dental casts exhibit minimal dimensional changes, retaining their form after setting. Dental plasters, made from a mineral composed of calcium sulfate dihydrate, form the basis of dental casts, providing a solid, durable structure for dental professionals to study and plan treatments effectively [5]. By converting plaster casts into digital models through 3D CT scans, dentists and dental technicians can perform precise measurements and analyses that are not possible with traditional manual work. Moreover, digitization allows for easy manipulation of the virtual 3D model, facilitating treatment planning and communication between dental professionals. The virtual models can be adjusted digitally to simulate different treatment options or to fabricate custom-made aligners or crowns [6]. However, there are some challenges associated with CT scanning of dental casts, such as scatter and beam-hardening artifacts [7]. These artifacts can significantly degrade the accuracy of the resulting 3D models [8]. 

In CT scanning, X-ray photons interact with the matter, resulting in absorption and scattering. The energy-dependent absorption of polychromatic X-ray photons leads to beam-hardening artifacts, commonly known as “cupping artifacts”, in the middle of a uniform object. The beam-hardening artifacts may cause CT number anomalies in dense materials (e.g., bone and dental enamel) and shades or streaks around highly attenuating objects [9,10,11,12]. The scattered X-ray photons also produce artifacts appearing as blurring, shades, and streaks. Particularly in 3D CT scans, the X-ray photon scattering increases as the scan volume increases. In 3D dental modeling from the CT images, these artifacts may introduce detrimental errors in the models, and the errors may impair the treatments. For example, inaccurate modeling of teeth and bone morphology may lead to ill-fitting restorations or improper implant placement. Therefore, it is required to reduce the modeling errors associated with scatter and beam-hardening artifacts in 3D modeling [13].

There are hardware-based and model-based methods for scatter artifact correction. An efficient hardware-based method is the use of a grid on top of the X-ray detector. Despite the effectiveness of grids in scatter artifact correction, the use of a grid inevitably decreases the signal-to-noise ratio (SNR) of the CT images due to the absorption of the primary (non-scattered) X-ray photons by the grid [14,15]. Meanwhile, the model-based scatter correction methods use mathematical models that predict the scattered photon distribution on the projection image based on the scan geometry and the material distribution in the object. A Monte Carlo simulation can be used to estimate the scattered photon distribution on the detector plane. However, it demands prior knowledge of the CT scan geometry and material distribution in the object, in addition to the extensive computational cost [16,17,18,19,20,21,22,23]. Other model-based methods use analytical approximation formulae to estimate the scattered photon distribution [24,25,26,27,28,29,30,31,32]. The estimated scatter distribution is then subtracted from the measured projection data to correct the scatter artifacts in the reconstructed CT images [32,33,34]. Ohnesorge et al. introduced a model-based method that has the advantages of low computational cost and flexibility to fit various scan geometries through simple parameter adjustments, even without prior knowledge of the material distribution in the object. However, the model-based method has a side effect, that is, an increase in the image noise, which complicates the beam-hardening artifact correction [32]. However, the model-based method is still suitable for artifact correction because dental casts have a homogeneous composition, i.e., dental plaster. Hence, the model error has less of an effect on the artifact correction than it does in the case of heterogeneous composition.

We have developed a new method that can effectively reduce both scatter and beam-hardening artifacts. We have modified the model-based scatter correction method proposed by Ohnesorge et al. and integrated it with the beam-hardening correction method [35] to minimize both artifacts in the CT images of plaster dental casts. We have also investigated the effects of artifact correction on the accuracy of the 3D digital models by comparing them with the optical scan-based 3D models.

## 2. Methods

### 2.1. Scatter Signal Estimation in the Projection Image

In order to reduce scatter artifacts in CT images, it is necessary to estimate the scatter signal from the raw projection data. The raw projection data, denoted as $P_{0}$, are defined as the negative logarithm of the flat field corrected projection intensity $I_{ffc}$. $I_{ffc}$ can be calculated by dividing the transmitted X-ray intensity by the incident X-ray (white field image) intensity. If there is X-ray scattering, the logarithm of the projection data is no longer the line integral of the attenuation coefficient along the scan line. Ohnesorge et al. used the following modification of the projection data for the scatter signal estimation [32]:

(1) $$P_{p}=K({I_{\varepsilon })}^{p}{\left(P_{0}\right)}^{q}$$

where *K* is the scatter correction factor and *p* and *q* are the adjustment parameters, which are determined depending on the shape and size of the objects. $I_{\varepsilon }$ is the projection intensity obtained by thresholding $I_{ffc}$:

(2) $$I_{\varepsilon }=\left\{\begin{array}{l} I_{ffc}\;\;\;\;\;\;\;\;\;\;\;\;\;\;\;\;\;\;\;\;I_{ffc}<\varepsilon \\ 0\;\;\;\;\;\;\;\;\;\;\;\;\;\;\;\;{\varepsilon \leq I}_{ffc}<1 \end{array}\right.$$

In dental CT imaging, longer or denser parts produce lower projection intensity; hence, the above equation takes account of the scan lines that are longer or denser than the preset value. By doing this, the noise amplification during the scatter correction can be alleviated. The thresholding step in the above equation is for determining which scan lines strongly contribute to the scatter artifact correction, typically focusing on high-density objects other than soft tissues, like metals, bones, or teeth. However, in the case of dental cast imaging, this step is not necessary since a dental cast is made of homogeneous material, i.e., dental plaster, and we can use the flat-field corrected projection intensity $\left(I_{ffc}\right)$ instead of $I_{\varepsilon }$ in Equation (1).

The scatter signal $P_{sc}$ on the projection image is estimated by convolving $P_{p}$ with a Gaussian scatter filter $G_{sc}$:

(3) $$P_{sc}={P_{p}*G}_{sc}.$$

We use a Gaussian filter $G_{sc}$, defined as follows:

(4) $$G_{sc}\left(x,y\right)=\frac{1}{2\sigma^{2}}e^{-\frac{x^{2}{+y}^{2}}{2\sigma^{2}}}$$

in which the kernel width σ is determined experimentally. The corrected projection data are then calculated by subtracting the estimated scatter signal from the raw projection data.

(5) $$P_{c}={P_{0}-P}_{sc}$$

In dental cast imaging, four parameters need to be adjusted to optimize scatter artifact correction. The first parameter, K, which determines the strength of the correction, should be selected in a way that avoids negative values in the corrected projection data. The two other parameters, p and q, both of which are one in the original modeling, serve as adjustment parameters to compensate for modeling errors. *p* and *q* can vary according to the scan parameters, such as tube voltage and magnification. Once these parameters have been determined for a specific imaging setup, the parameters remain constant. Lastly, σ controls the width of the Gaussian kernel, i.e., the dispersion of the scatter signal. 

### 2.2. Combined Algorithm (Beam-Hardening and Scatter Artifact Reduction)

To reduce the cupping artifacts caused by the X-ray beam hardening in dental cast imaging, we have developed a combined algorithm that simultaneously corrects both scatter and beam-hardening artifacts. We initiate the process by applying the DSC algorithm used in our previous work [35] to the raw projection data.

(6) $$P_{DSC}=\frac{1}{2\lambda }(e^{\lambda P_{0}}-e^{-\lambda P_{0}})$$

**Figure 1:**
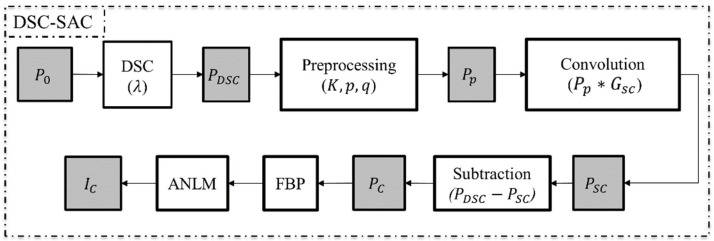
The beam-hardening and scatter artifact correction (DSC-SAC) algorithm.

The DSC algorithm requires a single parameter (λ) that controls the beam-hardening correction (BHC) power. After applying the DSC algorithm, we applied the scatter artifact correction (SAC) to the DSC-processed projection data $\left(P_{DSC}\right)$. Then, the CT images were reconstructed using a filtered back projection algorithm (FBP). To eliminate the noise that may be induced after correction, we applied the adaptive non-local mean (ANLM) filter to the CT images. The detailed steps of this combined algorithm are summarized in the flowchart presented in Figure 1.

### 2.3. Evaluation Phantoms

**Figure 2:**
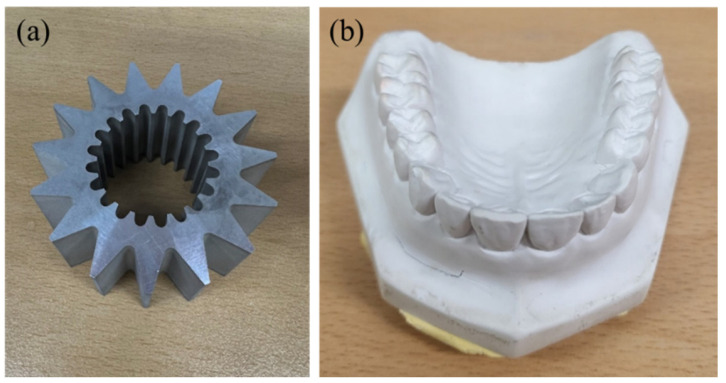
The phantoms used for the evaluation of the beam-hardening and scatter artifact correction. (**a**) The aluminum gear and (**b**) the dental cast.

Experiments were conducted using two phantoms to evaluate the performance of the DSC-SAC algorithm. The first phantom, shown in Figure 2a, was an aluminum gear with 32 mm in height and 52 mm in external diameter. The second phantom employed was the dental cast derived from a dental impression of human teeth, as illustrated in Figure 2b. We used a micro-CT called RAYDENT Microscan (Ray, Seongnam, Republic of Korea) to obtain the projection data of the phantoms. The micro-CT system was equipped with a 1256 × 1256 flat panel detector with a 119 µm pixel pitch and an X-ray tube with a 40 µm focal spot. The tube voltage and current could be adjusted in the range of 50–80 kV, and 0.4–0.7 mA. The projection data of dental plaster casts were acquired at 80 kV and 0.7 mA. The pixel binning was set by a factor of two, resulting in projection image sizes of 628 × 628, with a number of projections of 720. The exposure time was 0.1 s, which led to a total scan time of 72 s. The magnification ratio of the micro-CT system was consistently set to 1.7:1, yielding a nominal spatial resolution of 160 µm for the CT images. The field of view (FOV) of the micro-CT system was 8.5 cm × 8.7 cm, and the reconstructed 3D image had a matrix size of 624 × 624 × 640.

## 3. Results

### 3.1. Scatter Artifact Correction

**Figure 3:**
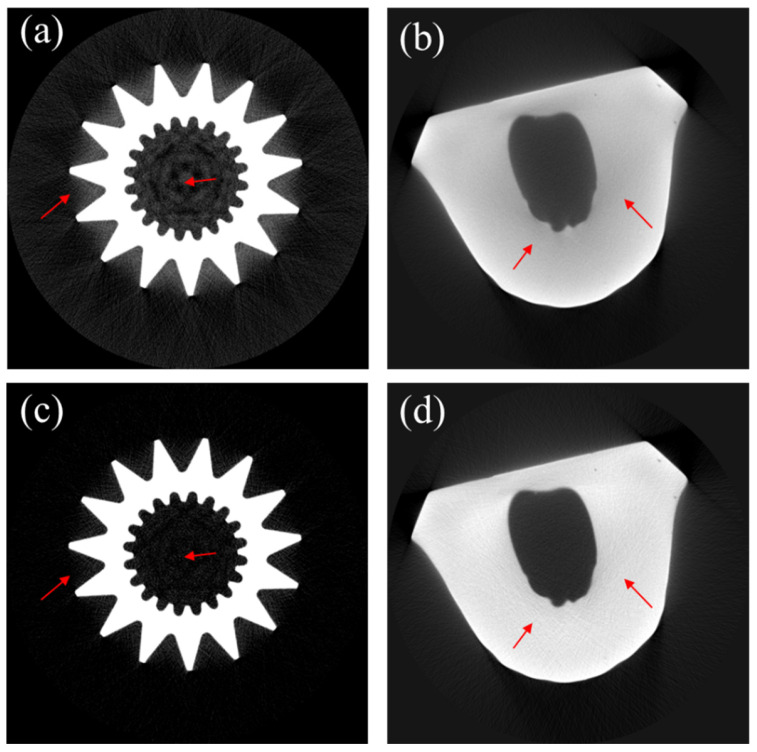
Scatter artifact correction of phantom images. (**a**,**b**) are the uncorrected CT images of the aluminum gear phantom and the dental cast, respectively. (**c**,**d**) are the corresponding CT images after scatter artifact correction.

Figure 3 shows CT images of the phantoms shown in Figure 2. Figure 3a,b show the uncorrected CT images of the aluminum gear phantom and the dental cast, respectively. Figure 3c,d are the corresponding CT images corrected with the scatter artifact correction (SAC) algorithm. The red arrows in Figure 3c,d highlight the discernible reduction in scatter artifacts. The corrected images exhibit greater uniformity, higher contrast, and sharper edges. The correction parameters were determined by a trial-and-error approach, with visual inspection of the resulting images of the phantoms. For both phantoms, *K* and *σ* were set to 0.3 and 70, respectively. The adjustment parameters *p* and *q* were set to 1.7 and 0.9, respectively, for both cases. 

### 3.2. Combination of Beam-Hardening and Scatter Artifact Correction

**Figure 4:**
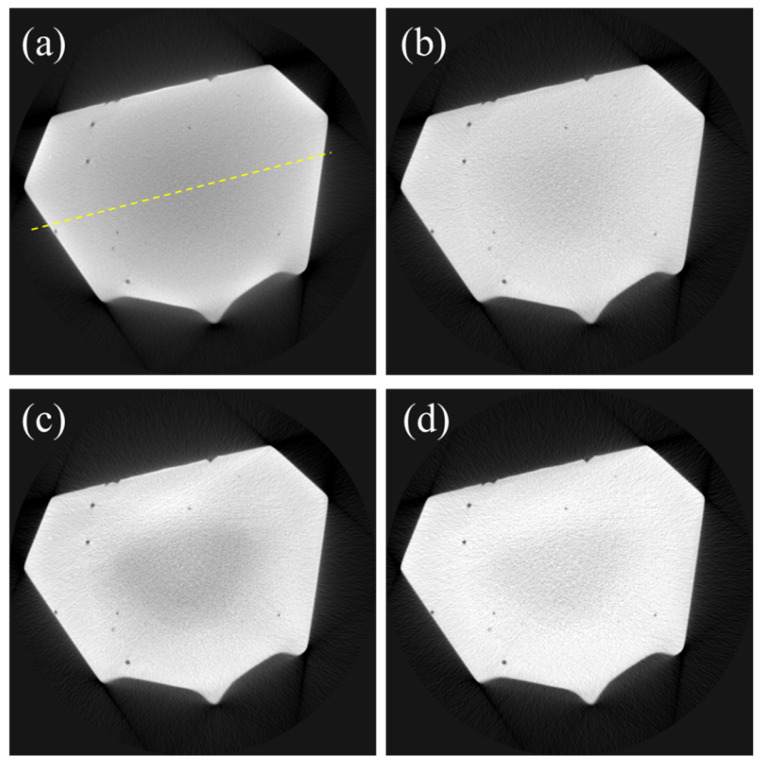
CT images of the base of the dental cast. (**a**) Uncorrected CT image, (**b**) DSC-based CT image, (**c**) SAC-based CT image, and (**d**) DSC-SAC-based CT image.

To assess and compare the performance of the combined algorithm in contrast to BHC only and SAC only, we applied the combined algorithm to the projection data of the dental cast. Figure 4a shows the uncorrected CT image, while Figure 4b–d show the CT images corrected by DSC, SAC, and the combined algorithm (DSC-SAC), respectively. The uncorrected image in Figure 4a has a prominent cupping artifact due to the heavy beam hardening in the base of the dental cast. Consequently, the DSC algorithm effectively reduced cupping artifacts to a greater extent than SAC. However, the application of the combined algorithm resulted in a significant reduction in cupping artifacts in addition to the improvement of image sharpness. For the dental cast images, the BHC correction parameter, λ, was set to 0.3, and the scatter correction parameters were set to *σ* = 70, *K* = 0.3, *p* = 1.7, and *q* = 0.9. For the combined algorithm, the parameters were set to *λ* = 0.3, *σ* = 70, *K* = 0.2, *p* = 1.7, and *q* = 0.9.

**Figure 5:**
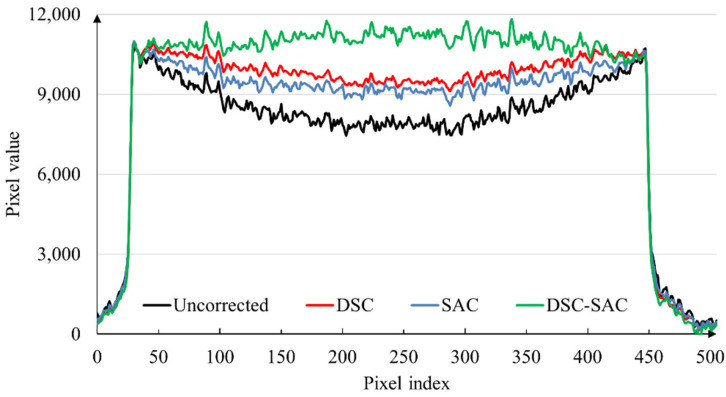
Line profiles along the yellow line shown in Figure 4a.

To show the performance in terms of reducing the cupping artifact in the dental cast image, we have included the line profiles in Figure 5 along the yellow line shown in Figure 4a. As can be seen from the line profiles, the DSC-SAC algorithm was most effective in reducing the cupping artifact. 

**Figure 6:**
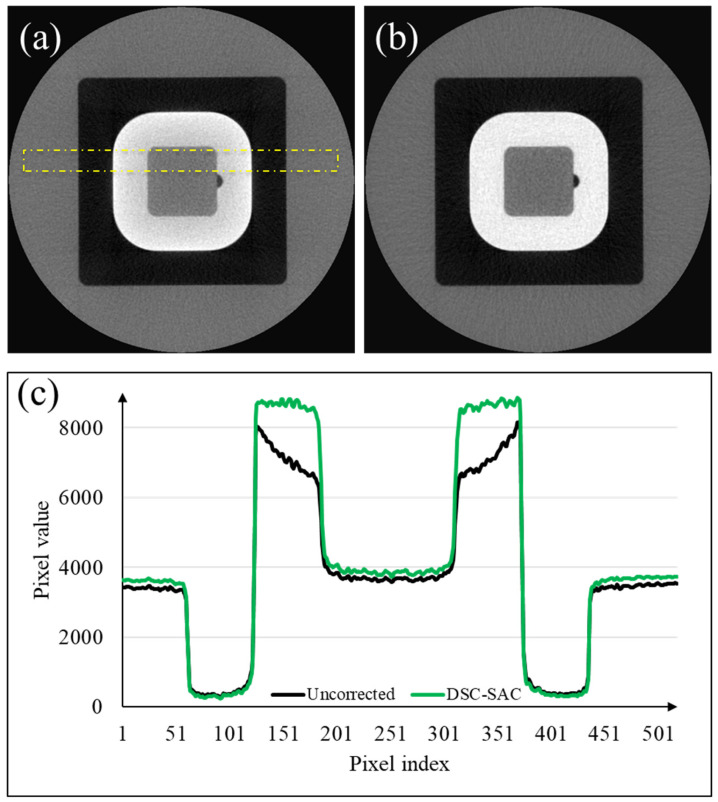
(**a**) Uncorrected CT image of the QUART phantom; (**b**) the corresponding image corrected by the DSC-SAC algorithm; and (**c**) the average line profiles in the yellow ROI shown in (**a**).

To quantitatively assess the performance of the combined algorithm, we used the quality assurance (QA) phantom (QUART DVT_KP, QUART GmbH, Zorneding, Germany). The phantom consists of two polymethylmethacrylate (PMMA) compartments (grey region in Figure 6a) with dimensions of 160 mm in outer diameter and 15 mm in height. In the middle of the phantom, a test object (white region in Figure 6a), polyvinyl chloride (PVC), was positioned surrounded by air (black region in Figure 6a). 

We measured the contrast (PVC to PMMA), noise (in the PMMA region), contrast-to-noise ratio (CNR), modulation transfer function (MTF), and image homogeneity from the images before and after the correction. The noise was measured using the standard deviation of the image intensity at a uniform ROI in the PMMA region. The CNR was the ratio of the contrast to the standard deviation in the PMMA region. The MTF was measured by positioning ROI over the PVC/air boundary where the largest signal drop-off appeared. In assessing image homogeneity, five regions of interest (ROIs) were selected within the PMMA area of the CT images. Subsequently, the mean intensity of each ROI was measured, and the maximum difference among the mean intensities of these five ROIs was then calculated. After normalizing the maximum difference by the average intensity across the ROIs, the homogeneity metric was computed as the ratio of the CNR to the normalized maximum difference. The measured performances are summarized in Table 1. Figure 6a,b show the CT images of the phantom before and after the correction, respectively. In addition, average line profiles are shown in Figure 6c, illustrating the effect of the correction algorithm on reducing cupping artifacts in the PVC region. 

**Figure 7:**
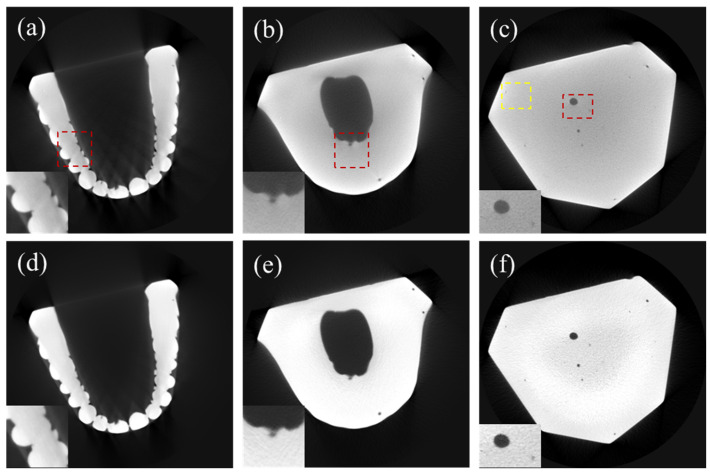
CT images of Model 1. (**a**–**c**) Uncorrected CT images. (**d**–**f**) DSC-SAC-corrected CT images. The zoomed images in the bottom left corner are related to the red ROIs while the yellow Roi is used to evaluate the signal to noise ratio (SNR).

We applied the DSC-SAC algorithm to the CT projection data of a few dental casts with different sizes and shapes. Figure 7a–c show the uncorrected CT images of a dental cast (Model 1), while Figure 7d–f show the corresponding corrected CT images. It is evident that the scatter artifacts, notably in the form of edge blurring, as well as the beam-hardening artifacts in the form of cupping artifacts, are strong in the original image. The combined algorithm effectively reduced both artifacts, particularly in the red ROIs highlighted in the corner. 

**Figure 8:**
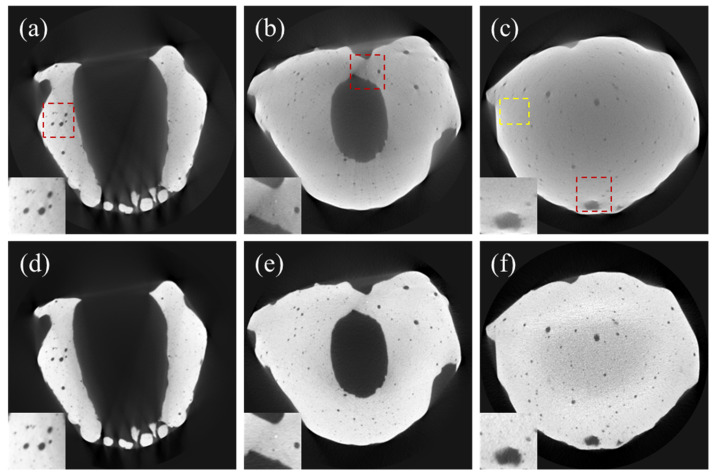
CT images of Model 2. (**a**–**c**) Uncorrected CT images. (**d**–**f**) DSC-SAC-corrected CT images. The zoomed images in the bottom left corner are related to the red ROIs while the yellow Roi is used to evaluate the signal to noise ratio (SNR).

**Figure 9:**
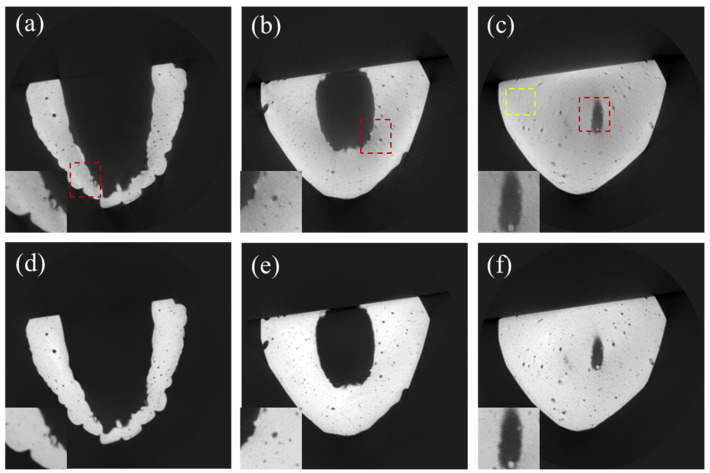
CT images of Model 3. (**a**–**c**) Uncorrected CT images. (**d**–**f**) DSC-SAC-corrected CT images. The zoomed images in the bottom left corner are related to the red ROIs while the yellow Roi is used to evaluate the signal to noise ratio (SNR).

Likewise, Model 2, as shown in Figure 8, exhibited severe scatter and beam-hardening artifacts. Upon applying the correction, these artifacts were noticeably reduced, as highlighted in the red ROIs. Figure 9 shows the correction results for Model 3, which was smaller than Models 1 and 2 in size and weight. Due to its smaller size and weight, the artifacts were not as severe as in the case of Models 1 and 2. Nonetheless, the combined algorithm visibly enhanced the image quality, as can be noticed from the figures.

We measured the mean and standard deviation at the yellow regions in Figure 7, Figure 8 and Figure 9 to evaluate SNR improvement. In the case of Model 1, SNR increased from 12.08 to 27.54 (127.9%). Similarly, for Model 2, the SNR increased from 10.43 to 21.65 (106.7%). In the case of Model 3, the SNR increased to a lesser degree, from 14.47 to 18.33 (26.67%). This implies that SNR deteriorated further due to the scatter and beam-hardening artifacts as the object size increased.

### 3.3. 3D Modeling Error Analysis

**Figure 10:**
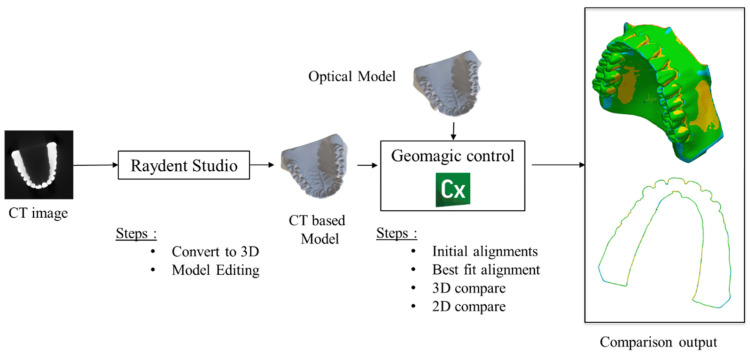
Analysis of 3D modeling errors between the CT-based model and the reference (optical) model.

To generate 3D STL data from the CT images, we used the STL generation tool named Raydent Studio (Ray, Seongnam, Republic of Korea) (http://www.raymedical.com.hk/raydent-studio/, 14 February 2024). Initially, we produced STL data covering the entire region of the dental cast, and subsequently, we manually cropped the region of interest, removing regions other than teeth and bones. The Raydent tool used global thresholding to segment the plaster region from the air region. First, a histogram of all pixels was calculated, and the optimal threshold was located between the peaks associated with air and plaster, typically falling within the range of 500–600 Hounsfield units (HU). In parallel, to create the ground truth (reference) STL data, we used a 3D optical scanner named Identica Hybrid (3D Wib, Busan, Republic of Korea). The optical scanner featured both mono and color cameras with resolutions of 1.3 M pixels and 5.0 M pixels, respectively. With a point spreading of 65 microns, it achieved fine precision in the scan. The volume coverage of the scanner was 80 mm × 60 mm × 60 mm. To facilitate the comparison between the reference STL data and CT-based STL data, we utilized the reverse engineering tool called Geomagic Control X (Artec 3D, Senningerberg, Luxembourg) (https://www.artec3d.com/3d-software/geomagic-control-x, 14 February 2024). Using this software, we computed deviation maps that displayed the Euclidean distance between the surfaces of the CT-based model and the reference model. With a sampling ratio of 100%, the numbers of points used to analyze the deviation between the STL data of the uncorrected CT model and the optical model were 159,513, 140,694, and 164,034 for Models 1, 2, and 3, respectively. Conversely, the numbers of points used for comparing the corrected CT model and optical model were 169,325, 142,273, and 162,030 for Models 1, 2, and 3, respectively. A summary of the comparison pipeline is shown in Figure 10.

**Figure 11:**
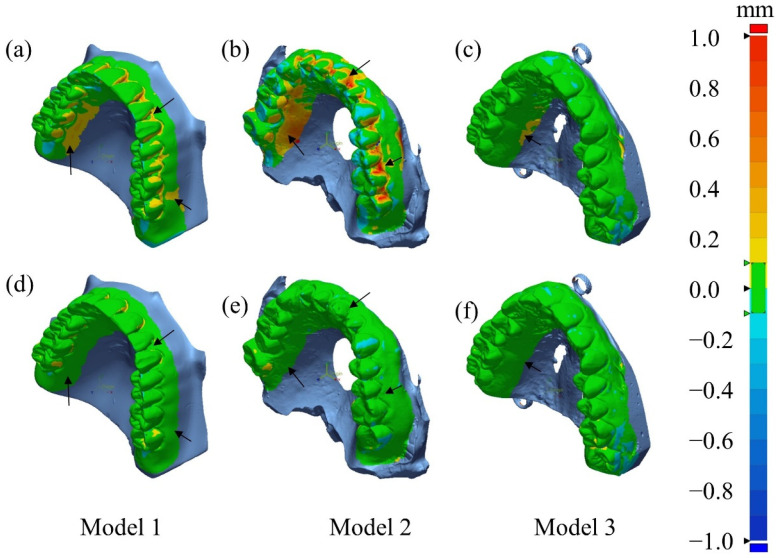
The 3D deviation maps of the CT-based STL data of three dental casts (Models 1–3). Deviation maps (**a**–**c**) for the uncorrected CT images and (**d**–**f**) for the corrected CT images. The accuracy of the 3D modeling was significantly improved after using DSC-SAC method as indicated with black arrows.

Figure 11 shows the deviations, relative to the optical 3D model, of the 3D models computed from the CT images of the dental casts. These deviations at each surface point on the 3D model signify the Euclidean distance from the corresponding surface point on the optical 3D model. The color maps presented in the first row (Figure 11a–c) and the second row (Figure 11d–f) show the deviations for the uncorrected CT images and the corrected CT images, respectively. In the cases of Models 1 and 2, significant improvements in 3D modeling accuracy are apparent in the regions indicated by the arrows. However, in the case of Model 3, smaller in size and weight than Models 1 and 2, the modeling error was not significantly different between the uncorrected and corrected cases. We have already noticed that the beam-hardening and scatter artifacts are not significant in the uncorrected CT images of Model 3, as can be noticed from Figure 9. 

**Figure 12:**
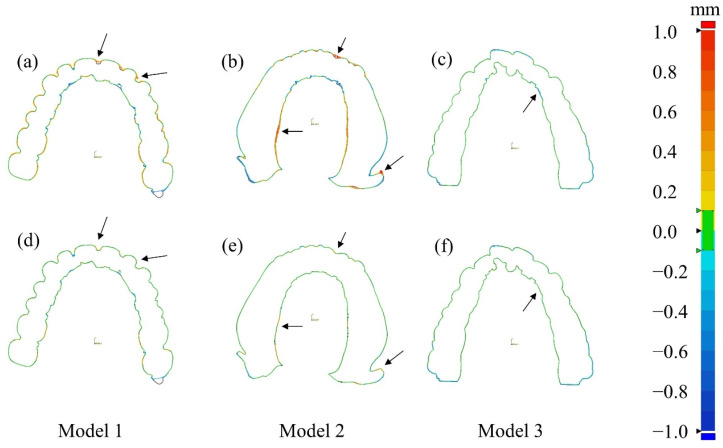
The 2D deviation maps of the CT-based STL data of three dental casts (Model 1–3). Deviation maps (**a**–**c**) for the uncorrected CT images and (**d**–**f**) for the corrected CT images. The accuracy of the 3D modeling was significantly improved after using DSC-SAC method as indicated with black arrows.

In addition to the 3D deviation maps shown in the previous figures, we show 2D cross-sections of the deviation maps in Figure 12 to provide a more detailed perspective on the accuracy of 3D models derived from CT images. This cross-section showcases the deviations relative to the reference 3D model, with similar color-coded representations as before. The observations from these cross-sections conform to the findings from the previous figures. In the cases of corrected CT images, particularly in larger models with more pronounced artifacts, we can make significant improvements in 3D model accuracy as compared to their uncorrected counterparts. However, for smaller models with fewer artifacts, the improvement in 3D model accuracy is less discernible.

We have also quantified the errors by measuring the root mean square (RMS) errors of the Euclidian distance. The RMS error can serve as an effective metric for assessing the overall deviations over the 3D deviation maps. The RMS values were reduced by 47.9% (from 0.102 to 0.053 mm), 71.7% (from 0.273 to 0.077 mm), and 8.8% (from 0.054 to 0.049 mm) for Models 1, 2, and 3, respectively. It is worth noting that the RMS value for Model 2 was relatively larger than other models. This discrepancy is attributed to the slightly larger size of Model 2 than Model 1 and 3.

## 4. Discussion

The primary objective of this study was to improve the spatial accuracy of 3D models derived from the CT images of dental casts. The scatter and beam-hardening artifacts in the CT images can cause significant spatial errors in the 3D models of dental casts, which may complicate dental treatment or prosthetic procedures. Most significant artifacts in the CT images of dental casts stem from X-ray beam hardening and X-ray photon scattering, and the artifacts usually appear in the form of shades, streaks, and edge blurring, all of which can induce spatial errors of the surfaces representing the bones and teeth. X-ray beam hardening can be avoided by using a monochromatic X-ray source, but it is not practical to use a monochromatic source for dental imaging. The scattering effect can be alleviated by employing grids on the X-ray detector. However, the use of grids inevitably increases X-ray exposure to the patient, apart from the rising cost of using grids. Therefore, reducing the artifacts by image processing is of great importance in expanding the use of dental CTs for digital dentistry. In this study, we combined the scatter artifact correction (SAC) and beam-hardening correction (BHC) algorithms to reduce the artifacts simultaneously. The experimental results demonstrate the efficacy of the combined algorithm in terms of many figures of merits, including CNR and MTF. 

In the 3D modeling of dental casts, the final figure of merits would be the spatial errors of the 3D model. As a reference 3D model, we used optical scan data of the dental casts, since optical scanning is recognized as a standard 3D modeling tool in modern digital dentistry. We showed the spatial error distribution on the deviation maps. It was noticed that the spatial errors were relatively small when the dental cast was small in size. We evaluated the overall spatial errors of the models by using the RMS measure. The RMS spatial errors of the 3D models were reduced by 47.9%, 71.7%, and 8.8% for Models 1, 2, and 3, respectively. 

The combined algorithm was implemented using CUDA on a personal computer equipped with an Intel^®^ Core™ i9-10850K processor, a GTX 1080 TI GPU, and 32 GB of RAM. The processing time for the computation of the combined algorithm and the 3D image reconstruction was approximately 11.5 s. The computation time included the reconstruction time of 5.3 s for a 624 × 624 × 640 3D image from projection data with the matrix size of 628 × 628 × 720. This computational efficiency signifies a possibility that the combined algorithm can be implemented in clinical settings for real-time or near-real-time applications.

Three-dimensional dental CT can be an alternative to intraoral optical scanning (IOS) for creating digital dental casts without taking conventional impressions. In modern digital dentistry, it is desired to skip the procedure of taking impressions for patient comfort and speedy treatment. Even though IOS is considered to have better spatial resolution than 3D dental CT, 3D dental CT has some advantages over IOS, i.e., no need for wide mouth opening, uniform coverage of deep-placed teeth, and no limitations in scanning concave regions. In this study, we scanned physical dental casts using a bench-top optical scanner and micro-CT system, and we demonstrated that the 3D digital model derived from the artifact-corrected CT images was nearly equivalent to the 3D model obtained from the optical scanner. However, we need further studies to compare digital models directly derived from CBCT scans or intraoral optical scans of patients without taking physical impressions. In a CT scan of a patient without a dental cast, other types of artifacts stemming from projection data truncation [36], wide cone beam angle [7], limited scan angle in a half scan [37], metal artifacts [35,38], and motion artifacts [39] should be addressed.

## 5. Conclusions

In conclusion, this study addressed a critical challenge in the use of 3D CT images for digital modeling by proposing an effective solution for reducing scatter and beam-hardening artifacts in the CT images of dental plaster casts. These artifacts have long been a concern in dental imaging, as they can compromise the accuracy and reliability of 3D dental modeling, which is crucial for treatment planning and custom prosthetic design. We combined the scatter artifact correction (SAC) algorithm and the beam-hardening correction (BHC) algorithm to reduce these artifacts in dental CT images simultaneously. We rigorously evaluated the accuracy of 3D models generated from corrected CT images, and we obtained a substantial improvement in the modeling accuracy, particularly in the case of larger dental casts. We expect that the proposed algorithm can be used by dental professionals who seek precise 3D modeling of dental casts for various applications, including implant placement, orthodontics, and prosthodontics. 

## Figures and Tables

**Table 1:** The performance measurement results for the QUART phantom.

Figure of Merit	Uncorrected	Corrected
Contrast	722.84	1091.25
CNR	12.25	23.5
Homogeneity	21.95	30
Noise in PMMA	33.79	27.33
MTF@10% (LP/mm)	2.18	2.42
MTF@50% (LP/mm)	1.12	1.12

## Data Availability

Data is unavailable due to privacy and ethical restrictions.
